# Long-term follow-up of pulmonary function in Fabry disease: A bi-center observational study

**DOI:** 10.1371/journal.pone.0180437

**Published:** 2017-07-25

**Authors:** Daniel P. Franzen, Albina Nowak, Sarah R. Haile, Dominique Mottet, Marco Bonani, Olivier Dormond, Malcolm Kohler, Pierre A. Krayenbuehl, Frederic Barbey

**Affiliations:** 1 Department of Pulmonology, University Hospital Zurich, Zurich, Switzerland; 2 Department of Internal Medicine, University Hospital Zurich, Zurich, Switzerland; 3 Epidemiology, Biostatistics and Prevention Institute, Epidemiology Department, University of Zurich, Zurich, Switzerland; 4 Department of Nephrology, University Hospital Zurich, Zurich, Switzerland; 5 Department of Visceral Surgery, Lausanne University Hospital, Lausanne, Switzerland; 6 Department of Internal Medicine, Linth Hospital, Uznach, Switzerland; 7 Transplantation Centre, Lausanne University Hospital, Lausanne, Switzerland; Baylor Health Care System, UNITED STATES

## Abstract

**Introduction:**

Fabry disease (FD) is a lysosomal storage disorder leading to decreased α-galactosidase A enzyme activity and subsequent abnormal accumulation of glycosphingolipids in various organs. Although histological evidence of lung involvement has been demonstrated, the functional impact of these changes is less clear.

**Materials and methods:**

Adult patients with FD who had yearly pulmonary function tests (PFT) at two centers from 1999 thru 2015 were eligible for this observational study. Primary outcome measures were the change in forced expiratory volume in the first second (FEV_1_) and FEV_1_/FVC over time. As secondary outcome we investigated sex, smoking, enzyme replacement therapy (ERT), residual enzyme activity, and Mainz Severity Score Index as possible predictors.

**Results:**

95 patients (41% male, 38.2 ± 14.5 years) were included. The overall prevalence of bronchial obstruction (BO, (FEV_1_/FVC < 70%)) was 46%, with male sex, age and smoking as significant predictors. FEV_1_ decreased 29 ml per year (95% CI -36, -22 ml, p<0.0001). FEV_1_ decline was significantly higher in males (p = 0.009) and in patients on ERT (p = 0.004). Conclusion: Pulmonary involvement seems to be a relevant manifestation of Fabry disease, and routine PFTs should therefore be included in the multidisciplinary follow-up of these patients.

## Introduction

Fabry disease is a rare X-linked lysosomal storage disorder due to mutations in the α-galactosidase A gene (*GLA)* leading to deficiency or absence of α-galactosidase A (α-GAL) enzyme activity. The enzymatic defect results in abnormal accumulation of neutral glycosphingolipids, particularly globotriaosylceramide and galactosylceramide, in the plasma and in tissue lysosomes throughout the body. The lysosomal dysfunction triggers inflammation and fibrosis. Essentially, the cells of the vascular endothelium are the main target of this disease. Thus, perfusion of kidneys, heart, nervous system, and skin can be impaired [1]. However, direct damage from deposits in other cell types may also contribute to organ dysfunction, such as e.g. airways and lung [2, 3].

The first patient with Fabry disease described by the German dermatologist Johannes Fabry in 1898 was suffering from “bronchial asthma” with frequent respiratory tract infections and died in his early forties because of respiratory impairment [4]. Subsequently, pulmonary involvement has been considered to be a leading cause of death in Fabry disease [5]. In a recent Fabry disease outcome survey, it was stated that pulmonary involvement was related to one of 42 deaths in Fabry patients, suggesting that it rather contributes to morbidity than to mortality [6]. However, while histological evidence of pulmonary involvement has been demonstrated [7–11], the functional impact of these changes is still a matter of debate [12, 13], and therefore pulmonary involvement in Fabry disease is not uniformly acknowledged by all researchers in this field [3]. Furthermore, the effect of enzyme replacement therapy (ERT) on pulmonary involvement is only limited to a few reports revealing that it may stabilize the functional decline of the lung [11, 14]. Above all, there is a paucity of data on serial pulmonary function tests (PFTs) in Fabry patients. Therefore, the exact mechanism of a possible lung involvement is widely unknown. The purpose of this study was to demonstrate the course of serial PFTs in a considerably large patient cohort with Fabry disease, to characterize the pathophysiological mechanism, and to identify patients at risk of pulmonary involvement and deterioration.

## Material and methods

### Patients

The University Hospitals of Zurich and Lausanne, Switzerland are conducting a shared cohort including patients with genetically proven Fabry disease. This cohort has been established in 1999 when ERT was in development. The patients have at least one yearly follow-up examination at one of the two study centers. Along with PFTs, the follow-up examination contains a comprehensive workup including medical history (including cigarette smoking history), clinical examination, complete blood and urine panel (including blood count, blood and urine chemistry), cardiology/angiology work-up (including electrocardiography, echocardiography and vessel sonography), nephrology work-up (including sonography of the kidneys), neurology work-up (including cerebral magnetic resonance imaging) and an ophthalmological examination. Indication for ERT is discussed at a three-monthly interdisciplinary conference with participation of all involved disciplines. According to local guidelines, ERT is indicated in all males. In females, ERT is indicated if they have proteinuria of more than 300 mg per day, Fabry-typical kidney biopsy findings, signs of Fabry-related cardiopathy such as left ventricular hypertrophy or arrhythmia, stroke or transient ischemic attack, acro-paraesthesia despite conventional analgesia, and/or gastrointestinal symptoms. ERT is prescribed at the licensed dose of either 0.2 mg/kg body weight of recombinant agalsidase-*α* (Replagal^®^) or 1 mg/kg body weight agalsidase-*β* (Fabrazyme^®^) and given intravenously every 14 days.

All adult patients of this cohort who were treated and followed-up at one of these centers from 1999 thru 2015 were eligible for this retrospective, multi-center observational study. Patients with less than two PFTs were excluded from the study. The study was approved by the local Committees of Ethics of both hospitals (Ethics committee of the canton of Vaude, Lausanne, Switzerland, protocol 101/01; and Ethics committee of the canton of Zurich, Switzerland, KEK-ZH 2012–0115), and is registered at ClinicalTrials.gov (Identifier: NCT01632111).

### Pulmonary function test

Yearly PFTs included spirometry and measurement of diffusing capacity of the lung for carbon monoxide (DLCO). Both tests were performed according to performance standards based on the statements from the American Thoracic Society (ATS) and the European Respiratory Society (ERS) [15, 16] at the Departments of Pulmonology of one of the centers. Bronchial obstruction was defined according to the Global Initiative for Chronic Obstructive Lung Disease (GOLD) [17] and ATS/ERS guidelines on chronic obstructive lung disease (COPD) [18] suggest referring the forced expiratory volume in the first second (FEV1) to the forced (expiratory) vital capacity (FVC) with a fixed cut-off value of below 70%. Values of DLCO were adjusted for the patient’s current hemoglobin value, and the patients were asked to withhold cigarette smoking at least four hours before PFT. Lung volumes and DLCO were measured with a commercial ZAN300 CO Diffusion system (nSpire Health GmbH, Oberthulba, Germany).

### Outcome measures

Primary outcome measures were the change in FEV_1_, FVC and DLCO over time, referring to the age of the patients. The secondary outcome measure was to investigate on possible predictors of pulmonary function changes over time. For this purpose, sex, cigarette smoking, ERT, residual α-GAL activity, and Mainz Severity Score Index (MSSI) were assessed. The latter is a clinical scoring system to determine the severity of Fabry disease, considering general, neurological, cardiovascular, and renal abnormalities [19].

### Statistical analysis

Patient characteristics at baseline were summarized as n (%) or mean (SD), and compared using a chi-square test or ANOVA, respectively. Changes in lung function were examined graphically, as well as with linear mixed models. We considered a random intercept and slope for each patient, as well as random intercept and slope for each center. However, the results were quite similar, and therefore we report only the models with patient-specific random effects. P-values less than 0.05 were considered statistically significant. All analyses were performed in R (version 3.2.2) R Core Team (2013).

## Results

**Fig 1:**
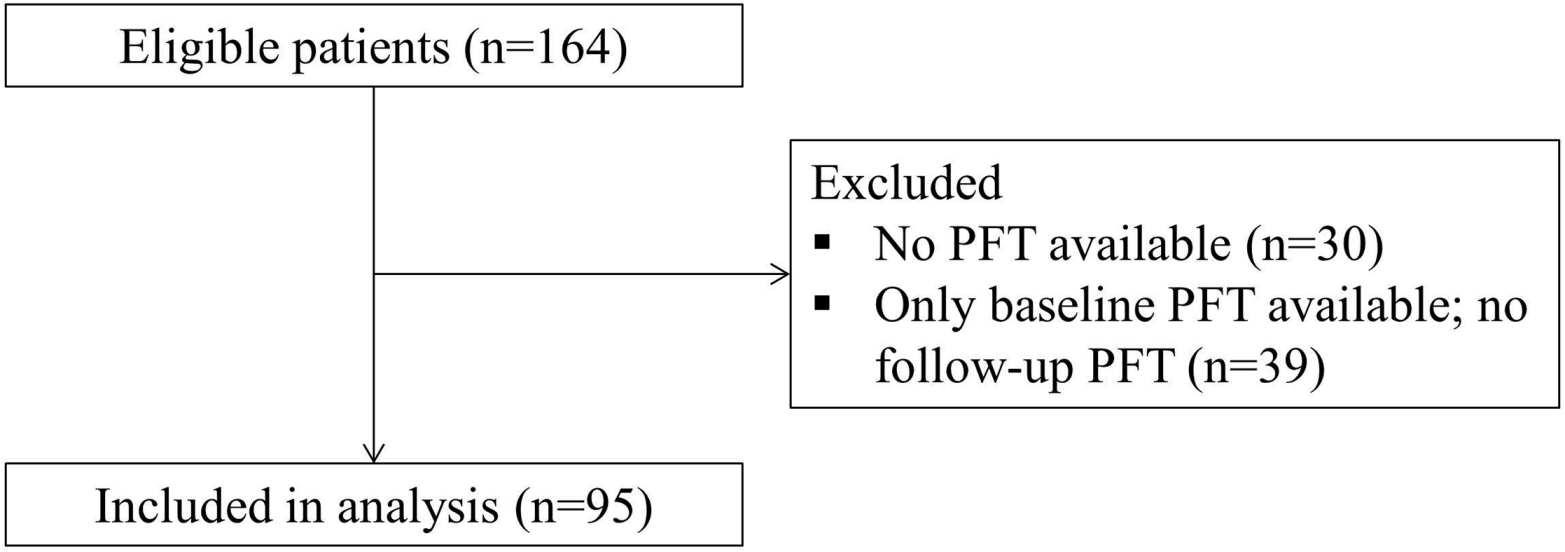
Patient flow chart. PFT, pulmonary function test.

From 164 eligible patients, 95 patients (59% male) with at least two PFTs were included in the analysis (Fig 1). Baseline characteristics are displayed in *Table 1*. At baseline, the mean patient age was 38.2 ± 14.5 years. Mean values of FEV_1_, FVC, and DLCO at baseline were above 80% predicted, and mean FEV_1_/FVC was above 70%. Thirteen patients (14%) had an obstructive lung disease. As expected, MSSI, residual α-GAL activity, and spirometric values were significantly different between both sexes (*Table 1*).

**Table 1:** Baseline patient characteristics, n = 95.

Variable	Male	Female	*p*-value	Total
	(n = 56)	(n = 39)		(n = 95)
Age, years	39.9 (14.0)	37.0 (14.8)	0.35	38.2 (14.5)
Age at ERT * begin, years	38.0 (14.7)	39.8 (14.8)	0.61	38.9 (14.7)
Body mass index, kg/m^2^	23.1 (3.9)	24.3 (4.8)	0.20	23.8 (4.5)
Current or former cigarette smoking	26 (72.2)	36 (67.9)	0.84	62 (69.7)
- Pack-years	21.3 (14.4)	12.1 (8.0)	0.04	15.7 (11.7)
MSSI ^ll^	23.1 (16.7)	11.2 (8.3)	<0.0001	16.1 (13.7)
Residual α-GAL ^†^ activity, %	8.6 (10.2)	53.2 (35.6)	<0.0001	41.5 (35.2)
FEV1 ^‡^, litres	2.9 (0.8)	2.5 (0.5)	0.0029	2.7 (0.7)
FEV1 ^‡^, % predicted	79.7 (18.2)	89.9 (16.1)	0.0052	85.7 (17.6)
FVC ^§^, litres	4.1 (0.8)	3.2 (0.6)	<0.0001	3.6 (0.8)
FVC ^§^, % predicted	91.6 (13.1)	99.1 (15.3)	0.02	96.1 (14.9)
FEV1/FVC, %	71.1 (11.2)	77.9 (7.8)	0.00075	75.1 (9.9)
DLCO **, % predicted	86.4 (20.3)	85.2 (12.1)	0.74	85.7 (15.6)

Values are presented as n (%) for categorical variables or mean (SD) for continuous variables.

^†^ α-GAL, α-galactosidase A activity

** DLCO, diffusing capacity of the lung for carbon monoxide

* ERT, enzyme replacement therapy

^‡^ FEV1, forced expiratory volume in the first second

^§^ FVC, forced (expiratory) vital capacity

^ll^ MSSI, Mainz Severity Score Index

### Changes of lung function over time

**Fig 2:**
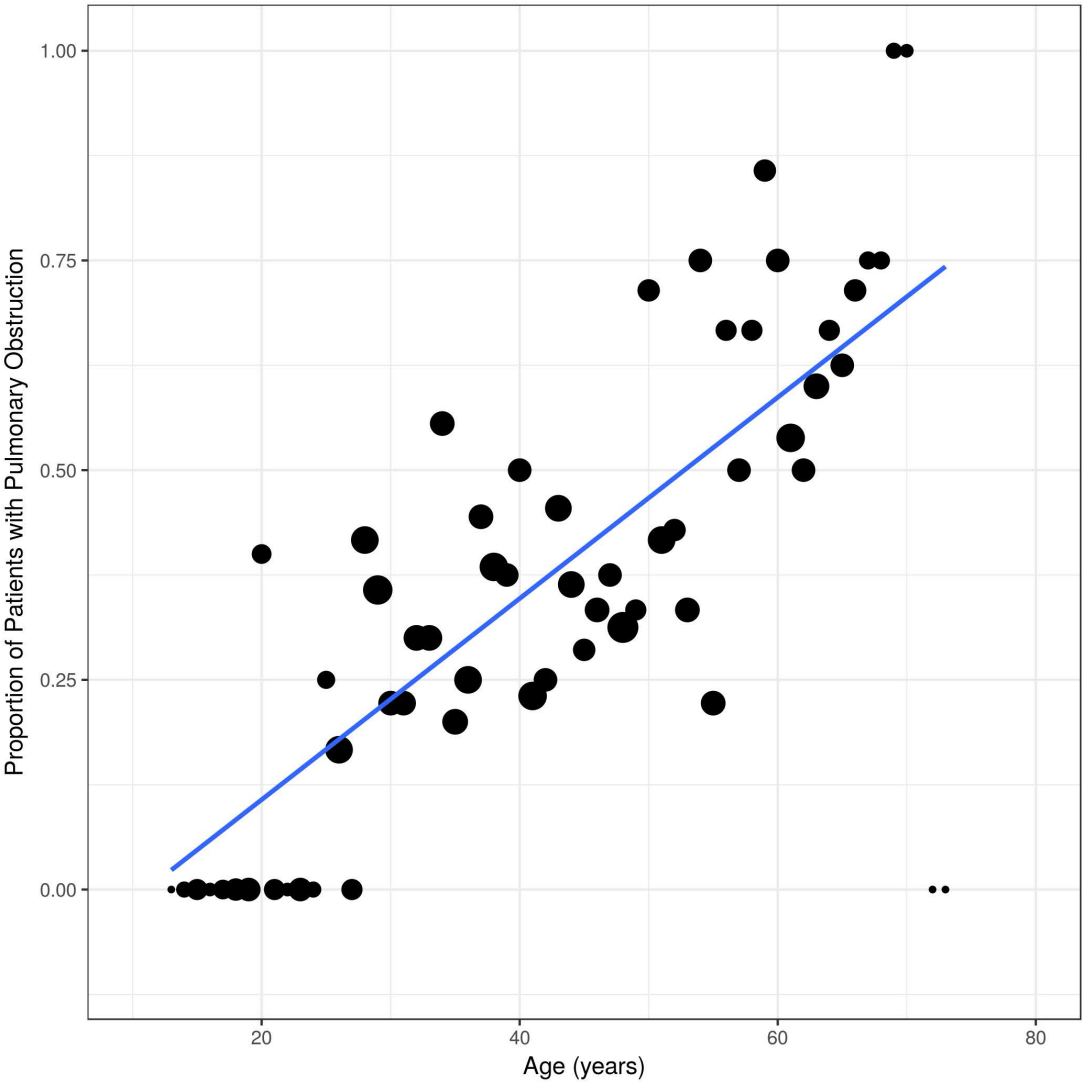
Proportion of Fabry patients with bronchial obstruction over time. All values on y-axis are presented as percentage of patients with bronchial obstruction which was defined as FEV1/FVC < 70%.

**Fig 3:**
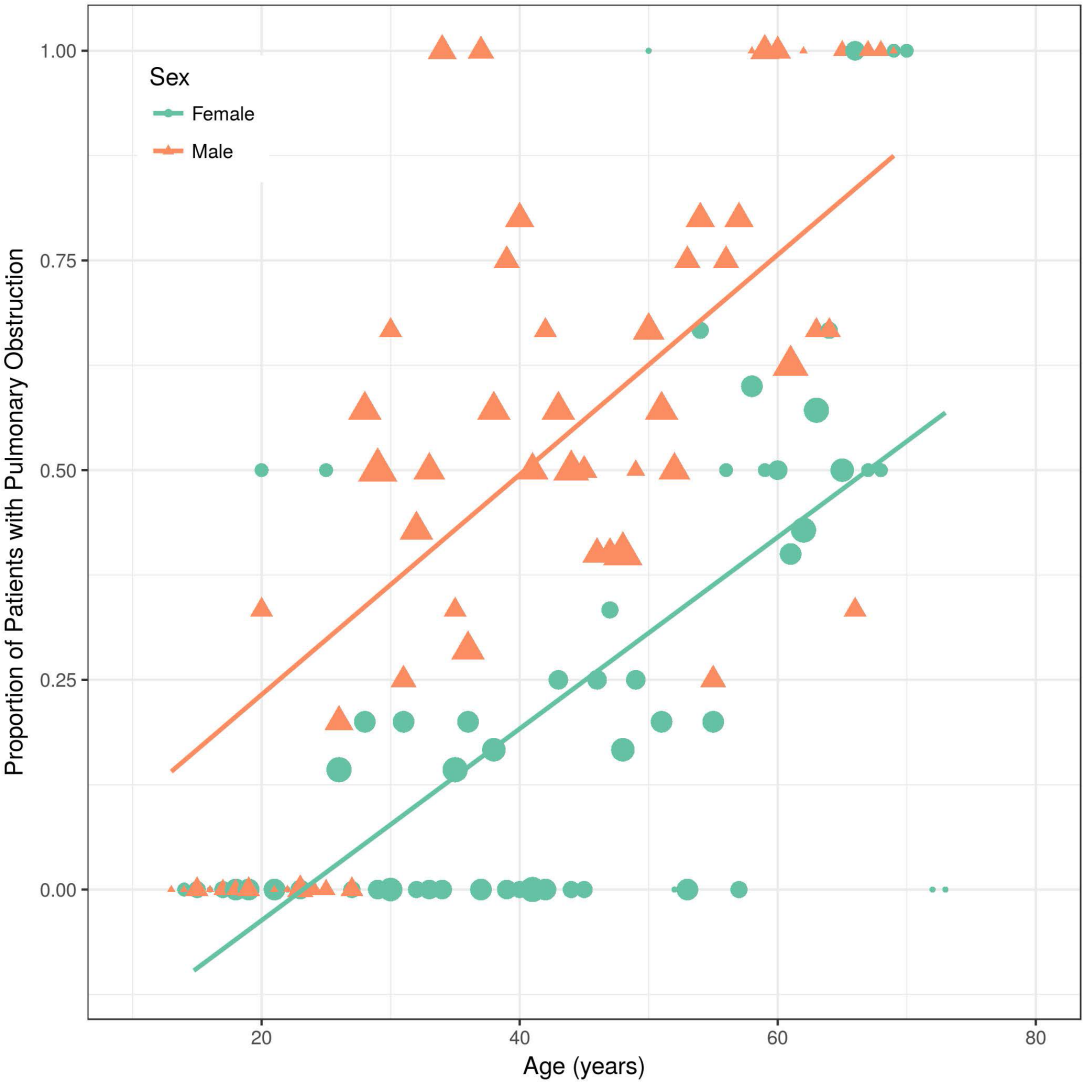
Proportion of Fabry patients with bronchial obstruction over time, divided by sex. All values on y-axis are presented as percentage of patients with bronchial obstruction which was defined as FEV1/FVC < 70%.

**Fig 4:**
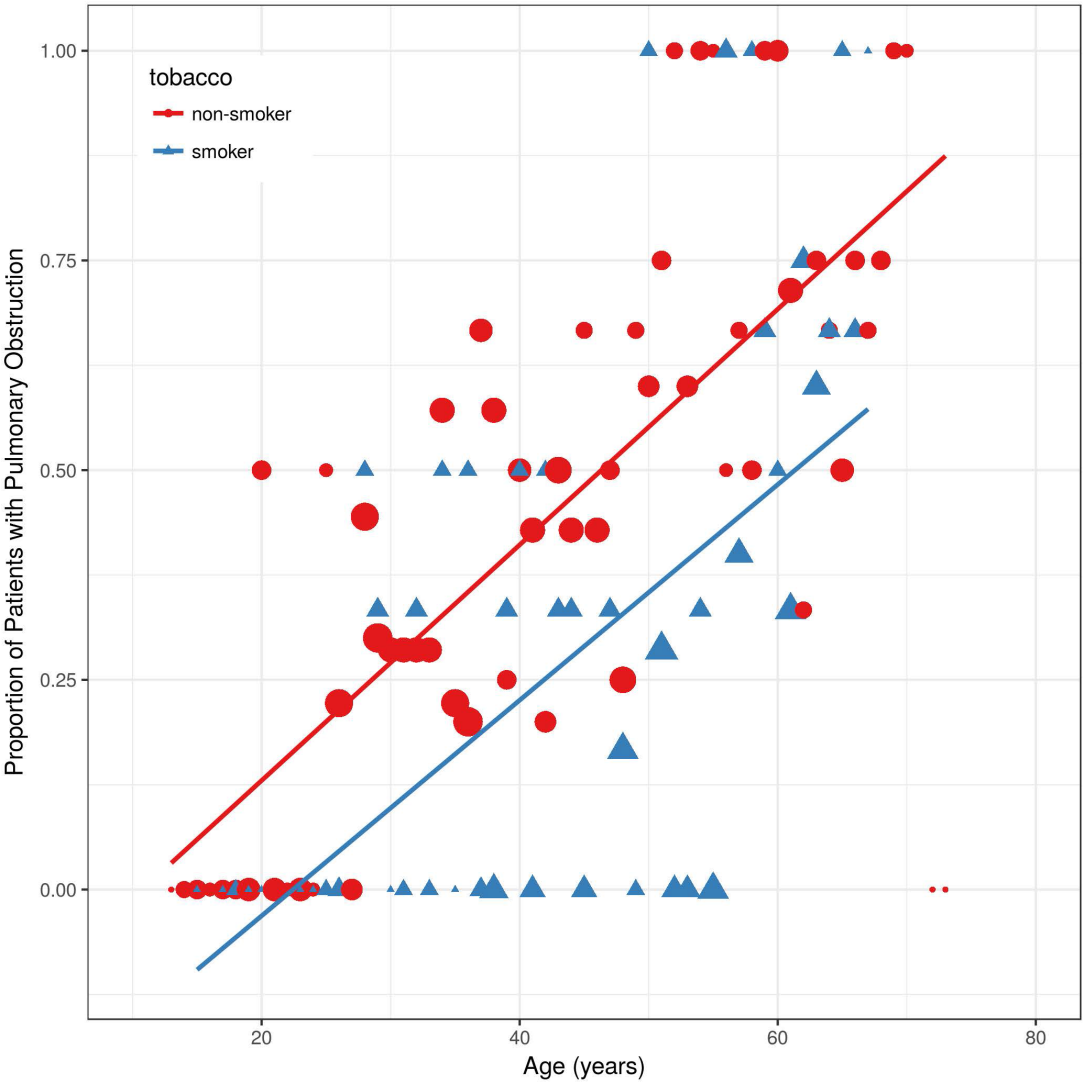
Proportion of Fabry patients with bronchial obstruction over time, divided by cigarette smoking. All values on y-axis are presented as percentage of patients with bronchial obstruction which was defined as FEV1/FVC < 70%.

Of the 82 non-obstructive patients at baseline, 31 developed bronchial obstruction over time (Fig 2). Thus, overall 44 patients (46%) had bronchial obstruction at any time point. As expected, the proportion of pulmonary obstruction in males and smokers was higher. Interestingly, the slopes of the curves were almost parallel in males compared to females (Fig 3), and in smokers compared to non-smokers, respectively (Fig 4).

**Fig 5:**
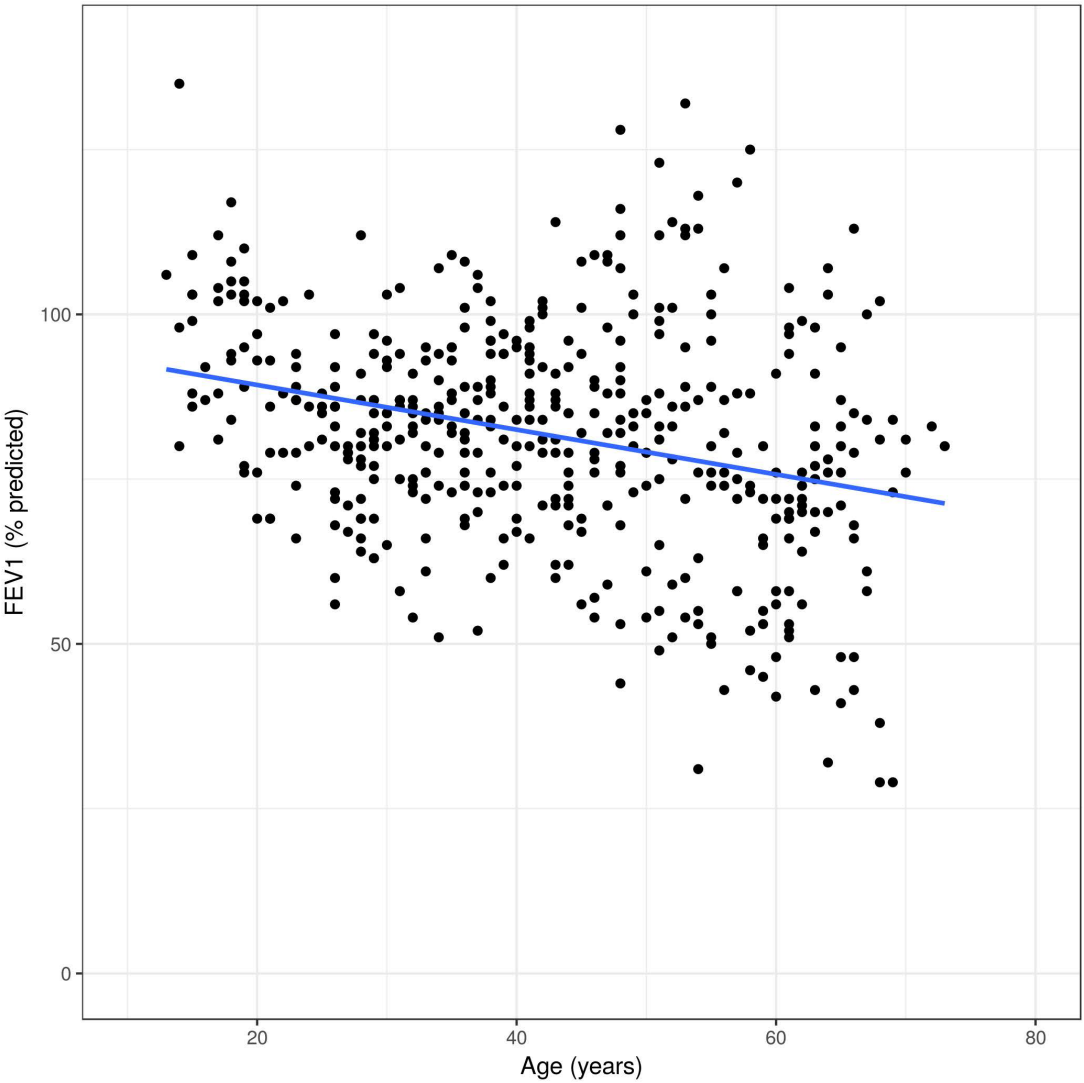
Unadjusted FEV_1_ changes in Fabry patients over time. All measures on y-axis are displayed in percent of the predicted values.

**Fig 6:**
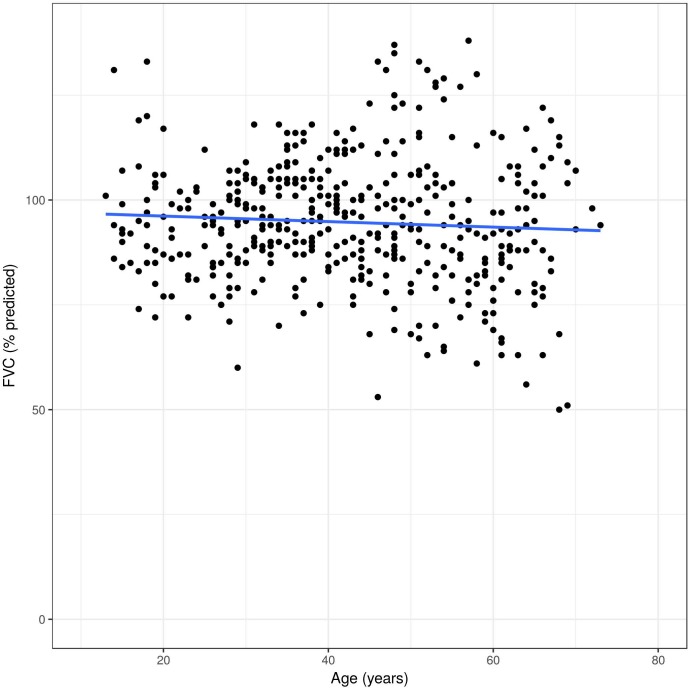
Unadjusted FVC changes in Fabry patients over time. All measures on y-axis are displayed in percent of the predicted values.

**Fig 7:**
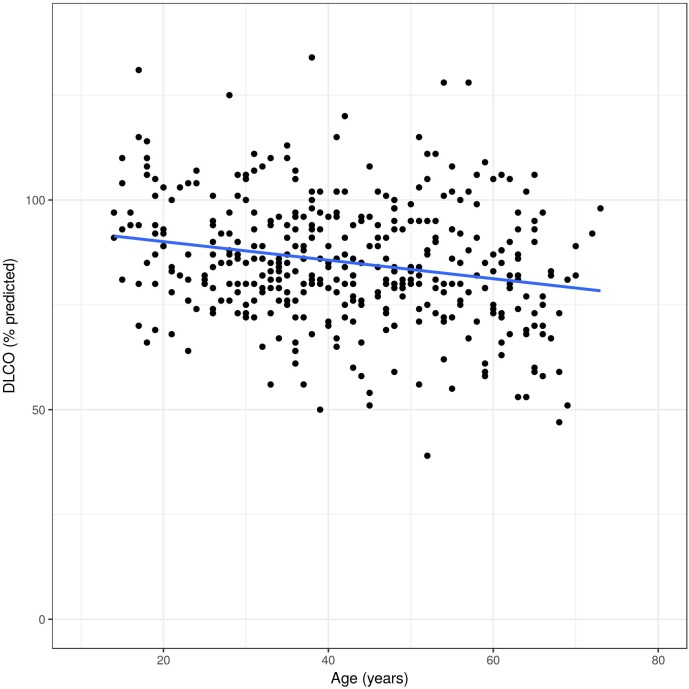
Unadjusted DLCO changes in Fabry patients over time. All measures on y-axis are displayed in percent of the predicted values.

Overall, FEV_1_ decreased significantly over time (-29 ml per year, 95% CI -36 to -22 ml, p<0.0001). Compared to this, FVC decreased -20 ml per year (95% CI -29 to -11 ml, p<0.0001), and DLCO remained almost unchanged over time (-0.14% predicted per year, 95% CI -0.32, 0.03% predicted, p = 0.11). The slopes over time of FEV1, FVC and DLCO in percent predicted values are illustrated in Fig 5, Fig 6 and Fig 7. The difference per additional year of age was -29 ml (95% CI -36, -22 ml, p<0.0001), and the reduced to -8 ml per year after adjusting for sex, smoking, MSSI, baseline FEV1, enzyme activity and ERT, (95% CI -13, -3 ml, p = 0.007).

### Risk factors for bronchial obstruction and FEV_1_ decline

In the univariable analysis age, male sex, cigarette smoking, MSSI, and ERT were associated with pulmonary obstruction. However, only age, male sex, and smoking remained significant when considered together (Table 2). The unadjusted odds ratio for each additional year of age was 1.23 (95% CI 1.09, 1.39, p = 0.0008), and almost the same even after adjusting for sex, smoking, MSSI, and ERT with 1.22 (95% CI 1.10, 1.34, p = 0.001).

**Table 2:** Results of generalized linear mixed models for pulmonary obstruction with random intercept and slope for each patient.

	Univariable analysis		Multivariable analysis	
	OR (95% CI)	p-value	OR (95% CI)	p-value
Age	1.23 (1.09–1.39)	0.0008	1.22 (1.08–1.39)	0.001
Male sex	86.80 (6.06–1242.78)	0.001	17.92 (1.28–251.64)	0.032
Smoking	51.80 (2.10–1277.70)	0.016	30.48 (1.58–586.72)	0.024
MSSI ^‡^	1.12 (1.00–1.25)	0.049	1.01 (0.92–1.10)	0.91
Residual α-GAL* activity	0.97 (0.93–1.01)	0.14		
ERT ^†^	451.18 (8.19–24956.63)	0.003	148.55 (0.80–27496.70)	0.06

* α-GAL, α-galactosidase A

^† ^ERT, enzyme replacement therapy

^‡ ^MSSI, Mainz Severity Score Index

FEV_1_ decline was significantly higher in male compared to female patients (p = 0.009), and in patients on ERT compared treatment-naïve patients (p = 0.004). Interestingly, FEV_1_ slopes did not vary significantly by smoking and MSSI (Table 3). Even when considering males only, there was no significant difference in FEV1 decline between smokers (-45ml (CI -65, -25ml) and non-smokers (-41ml (CI -55, -27ml)), p = 0.67.

**Table 3:** Comparative FEV_1_ slopes.

	Slope FEV1*, ml (CI)	*p*-value
Male	-38 (-48, -27)	0.009
Female	-20 (-30, -11)	
Smoker	-33 (-47, -19)	
Non-smoker	-29 (-38, -20)	0.60
ERT^†^	-31 (-39, -24)	
ERT^†^ naive	23 (-32, -14)	0.004
MSSI^‡^, 0–20 points	-28 (-38, -17)	
MSSI^‡^, 20–40 points	-31 (-46, -15)	0.33
MSSI^‡^, 40–60 points	-41 (-73, -10)	

^† ^ERT, enzyme replacement therapy

* FEV_1_, forced expiratory volume in first second

^‡ ^MSSI, Mainz Severity Score Index

## Discussion

To date, this is the largest study analyzing the long-term follow-up of pulmonary function in Fabry disease patients. Previously, the largest cohort study on pulmonary involvement in Fabry disease was published by Brown et al. describing 25 male Fabry disease patients of whom 36% were found to have bronchial obstruction [20]. In a recent review article by our group including 272 patients drawn from all reports on pulmonary involvement of Fabry disease, the prevalence of obstructive lung disease was 32% [3]. In the present study, the prevalence of bronchial obstruction was 46%. Notably, the prevalence of airflow obstruction in the general random population in a comparable age group in Switzerland is estimated 0.9% in women and 3.4% in men [21]. Yet, the prevalence of COPD in the general population ranges between 5.1% and 16.7% in women, and 8.5% and 22.2% in men [22]. Thus, our results underline a probably causative relationship between Fabry disease and bronchial obstruction, although no conclusion concerning the pathological mechanism is permissible based on our findings, since no lung biopsies had been taken. Yet, there are several reports suggesting that the progressive airway narrowing is due to accumulated glycosphingolipids in the peribronchial or peribronchiolar cells [8–11, 20]. According to the mechanism of endothelial dysfunction in cardiomyocytes and arterial smooth-muscle cells in Fabry patients, Wang et al. suggested that the pulmonary lysosomal accumulation of glycosphingolipids possibly leads to small and medium airway disease via hyperplasia of the bronchial/bronchiolar smooth muscle cells and via insufficient smooth-muscle relaxation [10]. Thus, particularly early in the disease process, glycosphingolipid accumulation in the bronchiolar smooth muscle cells in Fabry disease may cause small airway disease [23, 24]. In line with this, increased residual volumes as a consequence of bronchiolar obstruction were observed in a cohort of Fabry patients without evidence of emphysema [20]. Since there are no reliable indicators of small airway disease in spirometry, and since we did not perform bodyplethysmographies in our patients, we are not able to confirm the latter finding.

Unexpectedly, the overall FEV_1_ decline in our cohort of 29 ml per year is milder compared to the findings of others who stated a nonlinear progression of obstructive lung disease in Fabry disease with a yearly FEV_1_ decline of 40 ml [24]. However, according to our findings, the decline was significantly faster in male patients compared to their female counterparts (38 vs. 20 ml). Interestingly, the FEV_1_ decline in our study is comparable to healthy non-smoking individuals with a reported decline of 30–33 ml per year [25, 26]. And, compared to healthy smokers with a FEV_1_ decline of 40–45 ml per year [25, 26] or COPD patients with 47–79 ml per year [27], the FEV_1_ decline in Fabry disease patients is less severe than previously assumed. However, it has to be taken into account that a subset of male patients in our study is clearly more severely affected by Fabry disease, mirroring that it is characterized by considerably heterogeneous phenotypes. FEV1 decline in those patients is exceeding 40 ml per year, but appropriate tools to prospectively identify these severely affected patients in consideration of pulmonary involvement are lacking. Accordingly, MSSI was not associated with FEV_1_ decline in this study.

Compared to others, the impact of smoking on FEV_1_ decline in Fabry disease seems to be less clear [20, 28]. A possible explanation for these conflicting results could be due to heterogeneity of the reported cohorts. The subjects included in the cohort by Aubert et al. [28] were younger compared to the cohort reported by Brown et al. [20].

Interestingly, FEV_1_ decline was not improved by ERT, though ERT seems to be an indicator of marked disease severity. A possible reason for this observation could be that the initiation of ERT was relatively late. Hence, a high proportion of the patients could already have had bronchial/bronchiolar glycosphingolipid depositions, and early initiation of ERT might have prevented pulmonary disease progression. Regarding the efficacy of ERT on heart and kidney involvement, there is a probable decelerating effect on disease progression, when started prior to the onset of fibrosis [29–31]. However, there are still a lot of open questions in terms of initiation and duration of ERT, dosage, and the clinical relevance of the various endpoints used in the trials [32]. Furthermore, there are only limited and conflicting data on the effect of ERT on pulmonary function. In two small retrospective studies, ERT has been reported to stabilize or even ameliorate the burden of pulmonary involvement in Fabry disease, mainly bronchial obstruction and/or sleep apnea [10, 14]. Opposed to this, in one small retrospective cross-sectional case series of 15 Fabry disease patients no significant lung function changes were observed in serial spirometries and DLCO measurements during a period of 15 months after initiation of ERT [33]. Our study was not specifically designed and powered to answer this question. Future prospective studies should include PFTs as pre-defined endpoints, and adjust the analysis for potential confounding factors (e.g. smoking) [3]. Further investigations should clarify whether early ERT initiation can preserve pulmonary function. Clinical trials are also warranted in order to elucidate if inhaled bronchodilators on top of ERT are effective in patients with Fabry disease-related pulmonary obstruction.

Several limitations of this study merit consideration. Firstly, we used the GOLD criterion that defines bronchial obstruction as a FEV1/FVC < 70% rather than z-scores, although the GOLD guidelines misidentify a relevant proportion of abnormal younger adults as normal [34, 35]. Thus, it is possible that the prevalence of bronchial obstruction in our cohort is even higher than reported. However, it was not possible ad re-calculate all data, since some of them are several years old, and, comparison to older references is more accurate by using the GOLD criterion. Secondly, interpretation of our findings concerning the therapy effects should take into account that our study is not randomized and lacks a placebo-controlled group.

## Conclusions

Pulmonary involvement seems to be a relevant, but underestimated manifestation of Fabry disease, and routine PFTs should therefore be included in the multidisciplinary follow-up of these patients. However, additional studies are needed to investigate appropriate tools to prospectively identify patients at risk for pulmonary involvement, and whether early diagnosis and treatment initiation may prevent pulmonary disease progression before the onset of advanced bronchial/bronchiolar changes due to glycosphingolipid depositions.

## Supporting information

S1 File (XLSX)
